# 2679. Assessment of Sexually Transmitted Infection Incidence, Screening and HIV PrEP Uptake Among US Veterans with Opioid Use Disorder in Long Island, New York

**DOI:** 10.1093/ofid/ofad500.2290

**Published:** 2023-11-27

**Authors:** Pronoma Srivastava, Viraj Modi, Audun J Lier

**Affiliations:** Stony Brook University Hospital, Stony Brook, NY; Northport VA Medical Center, Northport, New York; Northport VA Medical Center, Northport, New York

## Abstract

**Background:**

Rates of sexually transmitted infections (STI) are on the rise in the US. Persons who inject drugs (PWID) and persons with opioid use disorder (OUD) are at an increased risk for acquisition of STI via high-risk sexual behavior concurrent with substance use. This study aims to compare rates of STI screening and diagnosis, hepatitis vaccination history, and receipt of HIV pre-exposure prophylaxis (PrEP) between PWID and non-PWID with OUD who presented to the Northport Veterans Affairs Medical Center (NVAMC).

**Methods:**

Data was collected from a retrospective chart review of US Veterans (USV) aged >18 years who presented to the NVAMC between 2010-2020 and carried an ICD9 or ICD10 diagnosis of OUD. Rates of STI screening and diagnosis, hepatitis A (HAV) and B (HBV) vaccination status, and receipt of PrEP were compared between PWID and non-PWID using either a two-sample T-test or Chi-square analysis.

**Results:**

We identified 502 USV with a diagnosis of OUD. Mean age was 52.6 years, 469 (92.4%) were male, 396 (78.9%) were white, 172 (34.8%) were employed and 216 (43%) had health insurance. A total of 337 (67.1%) USV had a history of cocaine use and 216 (43%) had a history of injection drug use. An STI was diagnosed in 51 (10%) USV, most frequently herpes simplex virus 1 or 2 (n=19, 3.8%), followed by syphilis (n=9, 1.8%). There was no difference in rates of STI between PWID and non-PWID USV. Eleven (2.2%) USV had HIV and 144 (28.7%) had HCV. A total of 411 (81.9%) USV received screening for HIV, 438 (87.3%) for HCV, 371 (74%) for syphilis, 160 (31.9%) for gonorrhea, and 169 (33.7%) for chlamydia. PWID were more likely to be screened for HIV (93.5% PWID vs. 73.1% non-PWID, p< 0.001), HCV (95.8% PWID vs. 80.8% non-PWID, p< 0.001) and syphilis (80.0% PWID vs. 69.2% non-PWID, p = 0.006) and to be vaccinated against HAV (73.6% PWID vs. 44.1% non-PWID, p< 0.001) and HBV (77.7% PWID vs. 54.3% non-PWID, p< 0.001). PrEP was prescribed in 4 (0.8%) USV.

**Figure:**
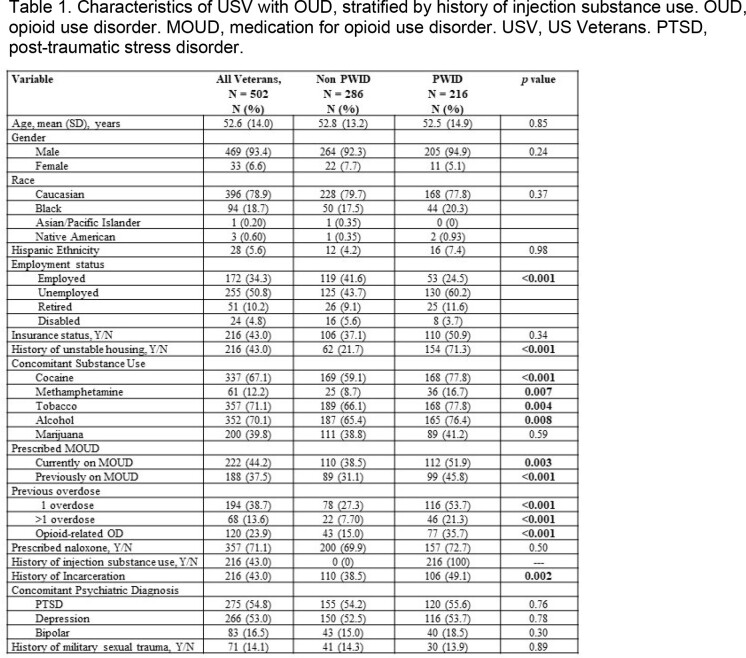

**Table 2.**
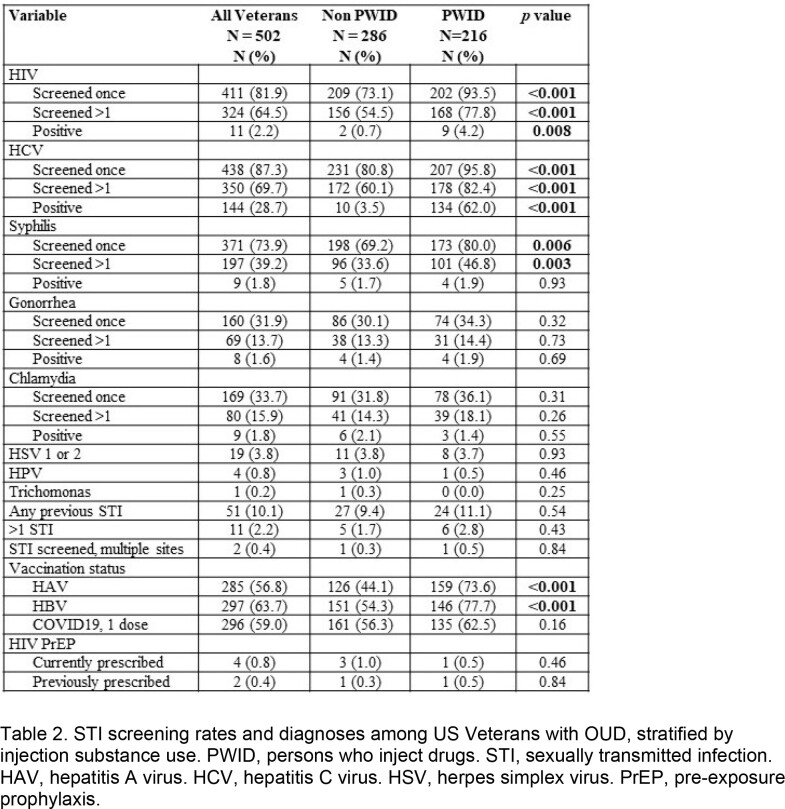
STI screening rates and diagnoses among US Veterans with OUD, stratified by injection substance use. PWID, persons who inject drugs. STI, sexually transmitted infection. HAV, hepatitis A virus. HCV, hepatitis C virus. HSV, herpes simplex virus. PrEP, pre-exposure prophylaxis.

**Figure 1.**
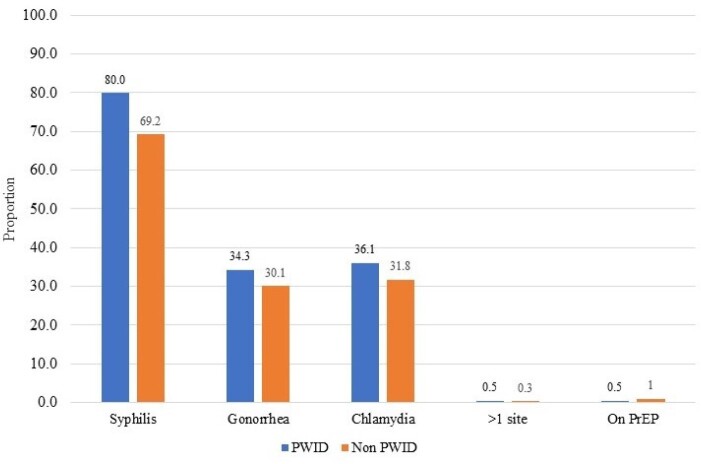
Rates of STI Screening among PWID and non PWID with OUD who presented to Northport VAMC. STI, sexually transmitted infection. PWID, persons who inject drugs. OUD, opioid use disorder.

**Conclusion:**

Among USV with OUD screening rates for gonorrhea and chlamydia occurred less frequently than for syphilis, HCV and HIV. PWID were more likely to be screened for HIV, HCV, and syphilis than non-PWID. There were low rates of PrEP uptake. USV with OUD may benefit from increased STI screening as well as linkage to PrEP evaluation and treatment.

**Disclosures:**

**All Authors**: No reported disclosures

